# Supplementary material for: Subgroup-Specific Associations of GRIA Genes Encoding AMPA Glutamate Receptor Subunits with Patient Survival in Medulloblastoma
Source: Neuromolecular Med. 2026 Jun 11;28(1):36. doi: 10.1007/s12017-026-08935-8 (PMC13260187; doi:10.1007/s12017-026-08935-8)
Supplement: Supplementary file 1 — Supplementary Material 1 [file 12017_2026_8935_MOESM1_ESM.pdf]

Supplementary information for

Subgroup-Specific Associations of *GRIA* Genes Encoding AMPA Glutamate Receptor Subunits with Patient Survival in Medulloblastoma

Bruno Saciloto • Matheus Dalmolin • Julia Caroline Marcolin • Martina Lichtenfels • Julia Vanini • Isabella B. Schröder Roesler • Jurandir M. Ribas Filho • Osvaldo Malafaia • Marcelo A.C. Fernandes • Caroline Brunetto de Farias • Amanda Thomaz • Rafael Roesler • Gustavo R. Isolan

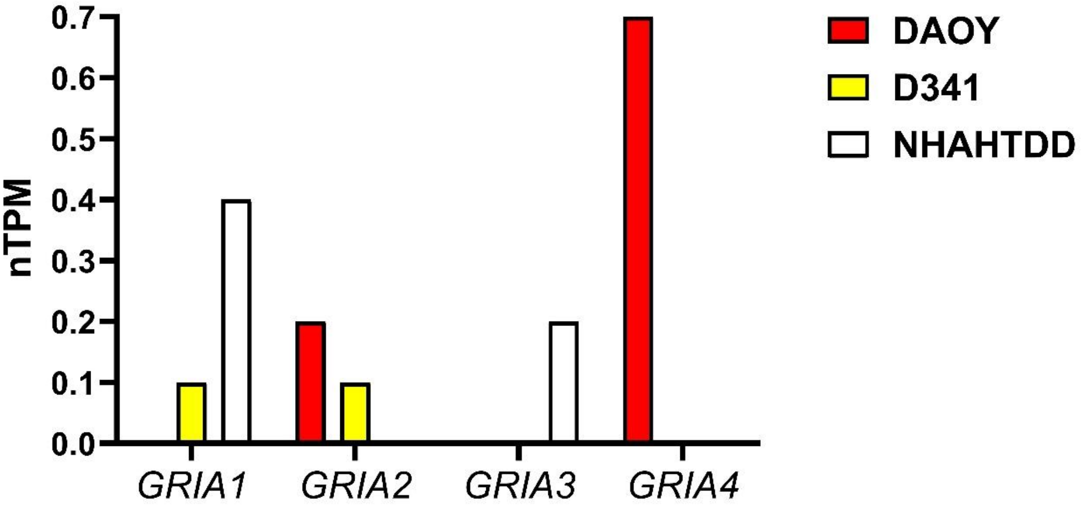

Supplementary Fig S1 Messenger RNA (expression of *GRIA* genes in DAOY MB, D341 MB, and NHAHTDD non-tumoral brain cells. Data normalized as transcript per million (nTMP) were obtained from The Human Protein Atlas (https://www.proteinatlas.org/; accessed on January 27th 2026).

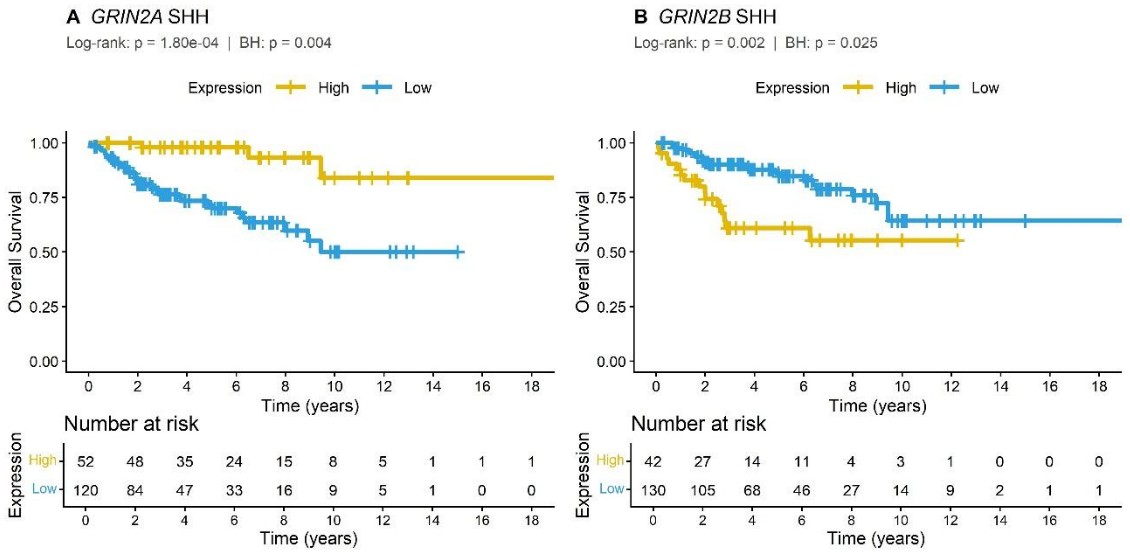

Supplementary Fig S2 Kaplan-Meier analysis of OS in patients bearing SHH MB tumors (n = 172) with higher or lower expression of the **A,** *GRIN2A*, and **B,** *GRIN2B* genes. Data were obtained from the dataset established by Cavalli et al. (2017). Log-rank

and adjusted *P* values are indicated in the panels. BH, Benjamini–Hochberg FDR correction for multiple hypothesis testing.

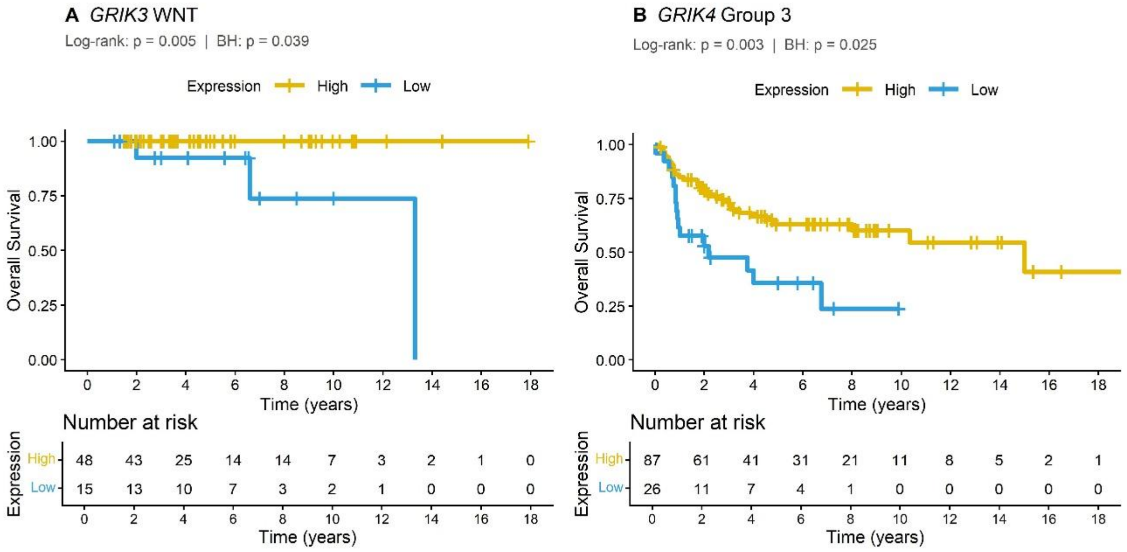

Supplementary Fig S3 Kaplan-Meier analysis of OS in patients bearing **A,** WNT MB tumors (n = 63) with higher or lower expression of the *GRIK3* gene, and **B,** Group 3 MB tumors (n = 113) with higher or lower expression of the *GRIK4* gene. Data were obtained from the dataset established by Cavalli et al. (2017). Log-rank and adjusted *P* values are indicated in the panels. BH, Benjamini–Hochberg FDR correction for multiple hypothesis testing.
